# A Randomized Pilot Trial of a Moderate Carbohydrate Diet Compared to a Very Low Carbohydrate Diet in Overweight or Obese Individuals with Type 2 Diabetes Mellitus or Prediabetes

**DOI:** 10.1371/journal.pone.0091027

**Published:** 2014-04-09

**Authors:** Laura R. Saslow, Sarah Kim, Jennifer J. Daubenmier, Judith T. Moskowitz, Stephen D. Phinney, Veronica Goldman, Elizabeth J. Murphy, Rachel M. Cox, Patricia Moran, Fredrick M. Hecht

**Affiliations:** 1 University of California San Francisco, San Francisco, California, United States of America; 2 UC Davis School of Medicine (Emeritus), Davis, California, United States of America; 3 Children's Hospital and Research Center, Oakland, California, United States of America; Indiana University Richard M. Fairbanks School of Public Health, United States of America

## Abstract

We compared the effects of two diets on glycated hemoglobin (HbA1c) and other health-related outcomes in overweight or obese adults with type 2 diabetes or prediabetes (HbA1c>6%). We randomized participants to either a medium carbohydrate, low fat, calorie-restricted, carbohydrate counting diet (MCCR) consistent with guidelines from the American Diabetes Association (n = 18) or a very low carbohydrate, high fat, non calorie-restricted diet whose goal was to induce nutritional ketosis (LCK, n = 16). We excluded participants receiving insulin; 74% were taking oral diabetes medications. Groups met for 13 sessions over 3 months and were taught diet information and psychological skills to promote behavior change and maintenance. At 3 months, mean HbA1c level was unchanged from baseline in the MCCR diet group, while it decreased 0.6% in the LCK group; there was a significant between group difference in HbA1c change favoring the LCK group (−0.6%, 95% CI, −1.1% to −0.03%, *p* = 0.04). Forty-four percent of the LCK group discontinued one or more diabetes medications, compared to 11% of the MCCR group (*p* = 0.03); 31% discontinued sulfonylureas in the LCK group, compared to 5% in the MCCR group (*p* = 0.05). The LCK group lost 5.5 kg vs. 2.6 kg lost in MCCR group (*p* = 0.09). Our results suggest that a very low carbohydrate diet coupled with skills to promote behavior change may improve glycemic control in type 2 diabetes while allowing decreases in diabetes medications.

This clinical trial was registered with ClinicalTrials.gov, number NCT01713764.

## Introduction

To optimize the health of individuals with type 2 diabetes, who suffer from the most costly chronic disease in the US [1], blood glucose control is important [2]. Although there is agreement that diet is an important strategy for treating type 2 diabetes, there is little consensus about the optimal diet. Current recommendations from the American Diabetes Association (ADA) suggest that “for weight loss, either low-carbohydrate, low-fat calorie-restricted, or Mediterranean diets may be effective” and that “the mix of carbohydrate, protein, and fat may be adjusted to meet the metabolic goals and individual preferences of the person with diabetes” [3]. However, the ADA's carbohydrate counting diet recommendations for persons with type 2 diabetes suggest consuming at least about 150 grams of carbohydrates per day [4]. Some research, on the other hand, has found that reducing carbohydrate intake even lower can improve glycemic control [5]. The evidence base for carbohydrate intake in type 2 diabetes has not been strong enough, however, to conclude whether lower carbohydrate diets are preferable for most persons with type 2 diabetes than the typical level in current standard diabetes diets. Limitations of prior studies include the lack of a randomized comparison group in many studies, poor retention, or a short duration of follow-up.

Our goal in this study was to conduct a randomized, controlled trial with good participant retention to compare the health impact of two different diets in type 2 diabetes over three months: (a) a medium carbohydrate, low fat, calorie-restricted, carbohydrate counting diet in line with guidelines from the ADA to (b) a very low carbohydrate, high fat, non calorie-restricted diet whose goal is to induce nutritional ketosis. Because participants who attempt to change their diets tend to revert to their original diet over time, reducing adherence and potentially retention, we included skills to support behavior change and maintenance [6], [7]. Although the current trial was aimed in part at comparing the effects of each diet on glycemic control, an important goal was to test the feasibility of our research design for conducting a larger scale, longer-term trial to more definitively address the limitations of prior research studies.

## Methods

### Ethics statement

This trial was reviewed and approved by the University of California, San Francisco (USCF) Institutional Review Board and registered with ClinicalTrials.gov, number NCT01713764. All participants provided written informed consent.

### Trial design

We performed a single site, parallel-group, balanced randomization (1∶1) trial in San Francisco, California (UCSF), which compared a moderate carbohydrate, calorie-restricted diet representative of conventional diabetic diet recommendations to a very low carbohydrate, ketogenic diet (≤50 g carbohydrates per day not including fiber) in persons with HbA1c>6.0%. The primary outcome measure was change in glycated hemoglobin (HbA1c) from baseline to 3 months. Key secondary outcomes were changes in lipids, insulin resistance as estimated by homeostatic model assessment (HOMA), and weight. The protocol for this trial and supporting CONSORT checklist are available as supporting information; see Checklist S1 and Protocol S1.

### Participants

To be eligible for inclusion in the study, participants needed to be aged 18 or over with a diagnosis of type 2 diabetes mellitus (HbA1c≥6.5) or prediabetes with an HbA1c above 6.0 [8]. Participants also needed to have a body mass index (BMI) of 25 or above, be willing to eat either diet, and have sufficient control over their food to follow the intervention instructions.

Exclusion criteria included: unable to provide informed consent; non-English speaking (groups were conducted in English); a substance abuse, mental health or medical condition that would, in the opinion of the investigators, make it difficult for the individual to take part in the intervention; current use of oral glucocorticoids or weight loss medications; pregnant or planning to get pregnant in the next 12 months; breastfeeding or less than 6 months postpartum; history of or planned weight loss surgery; vegan; or unwilling to do home glucose monitoring. We also excluded participants who were currently using insulin or taking more than three oral hypoglycemic medications to limit the complexity of potential medication adjustments needed to address the effects of diet changes on glucose levels.

### Recruitment and Randomization

Participants were recruited from the community and from local health organizations through advertising and announcements. They were paid $25 for completing the assessments at 3 months after intervention initiation. Eligible participants provided written, informed consent and were randomly assigned following block randomization procedures (computerized random numbers in blocks of four) to either the MCCR or LCK diet created by the first author. An assistant, not associated with any other aspect of the research, concealed the sequence in opaque envelopes, which were opened by other research assistants unaware of the sequence. For this trial, it was not possible for the participants and staff to be blinded to group allocation. To reduce potential intervention contamination if two related pairs of participants (a mother and a daughter, two sisters) were assigned to different groups, each pair was assigned as a unit, using pre-specified procedures for participant pairs; this was done for two pairs.

### Intervention

Participants attended 13 2-hour classes that met weekly at the UCSF Osher Center during most of the intervention period. One hour was devoted to instruction on their assigned diet, with three classes also discussing the importance of sleep and exercise. Each class session included a break during which snacks were provided appropriate to the assigned diet. Participants were encouraged to change their diet gradually; ideally, by the fourth class, participants were to have changed all of their meals to be in alignment with the new recommendations.

Half of each two-hour class in both groups was focused on learning skills to support behavior change and diet maintenance. Topics were drawn from two prior behavioral intervention models for diet and health behavior change, including components aimed at increasing positive affect and decreasing depressive symptoms [6] and teaching mindful eating techniques [7]. Specific topics included: setting attainable goals; scheduling, noticing, and savoring positive events; developing self-compassion; practicing positive reappraisal, gratitude, and acts of kindness; being aware of one's personal strengths; and being mindful of hunger, fullness, cravings, taste satisfaction, triggers for overeating, thoughts and emotions. To develop mindful eating skills, participants were asked to practice a guided meditation 10 minutes per day at least three times a week using audio CDs recorded for the intervention, and to use several mindful eating practices during meal times, such as focusing awareness on the taste and texture of foods while eating. The psychological skills training hour was led by a psychologist with experience teaching mindfulness and health behavior change.

#### MCCR Diet

Participants in this group were asked to follow a medium carbohydrate, low fat, calorie-restricted, carbohydrate counting diet consistent with guidelines from ADA, here referred to as MCCR (moderate carbohydrate, calorie-restricted). The MCCR diet classes were taught by a registered dietician with several years of diabetes education experience (author RC), using information from the *American Diabetes Association Complete Guide to Diabetes* [9], supplemented by several additional resources [4], [10]. Participants in the MCCR group were encouraged to derive 45% to 50% of their calories from carbohydrates and were taught to count carbohydrates using 15 grams of carbohydrates as a unit. We provided specific suggestions for the amount of carbohydrate units participants should eat at each of 3 meals and 2 snacks. Most participants were asked to eat 3 carbohydrate units per meal and 1 per snack, or roughly 165 grams of carbohydrates a day. They were also instructed to keep their protein levels about the same as before they started the study and to lower their fat consumption. We further recommended that participants eat 500 fewer kilocalories (kcal) per day than their calculated maintenance needs based on their age, weight, height, and physical activity level (ranging from sedentary to very active), using the formula from the Institute of Medicine Dietary Reference Guidelines [11].

#### LCK Diet

Participants in this group were asked to follow a very low carbohydrate, high fat, non calorie-restricted diet whose goal is to induce a low level of ketosis, here referred to as LCK (low carbohydrate, ketogenic). The LCK diet classes were taught by an author of the manuscript (LS), who is experienced in using the low carbohydrate dietary approach. Participants in the LCK group were encouraged to reduce carbohydrate intake over 7–10 days to between 20–50 grams of carbohydrates a day, not including fiber (referred to as net grams of carbohydrates), with the goal of achieving nutritional ketosis, defined as a blood beta-hydroxybutyrate level between 0.5 and 3 mM, as measured twice a week at home using blood ketone test strips with an Abbott Precision Xtra Monitoring System (Abbott Diabetes Care, Alameda, California). For example, if eating 50 net grams of carbohydrates led to a ketone level of 0.1, we instructed participants to lower their carbohydrate consumption. Participants were encouraged to eat a normal amount of protein (to keep their protein levels as they were before the intervention began, as long as they were meeting the minimum amount suggested according to the Institute of Medicine) and to derive their remaining calories from fat. The overall rationale informing the goal of achieving a low level of detectable blood ketones was that some of the benefits of a very low carbohydrate diet in type 2 diabetes may occur when carbohydrate intake is reduced to a level at which it no longer provides a strong stimulus for insulin release. An important function of insulin is to inhibit lipolysis and reduce levels of plasma non-esterified fatty acids, switching the main fuel source away from fatty acids and toward carbohydrates [12]. Nutritional ketosis thus serves as a marker indicating that insulin levels are reduced to a level that lipolysis is not inhibited. As the level of carbohydrate intake that is needed to release inhibition of lipolysis varies between individuals, monitoring nutritional ketosis potentially provides an individualized marker for titrating carbohydrate restriction. Nutritional ketosis may serve as a particularly relevant marker of carbohydrate restriction in the context of diabetes because it indicates a shift away from reliance on glucose as a primary energy source to fatty acids and ketones. As carbohydrates lead to elevated glucose levels in diabetes, fatty acids provide an alternative energy source that can provide adequate fuel without elevating glucose levels in the same way as carbohydrate. This level of “nutritional ketosis” is safe and physiologically different from ketoacidosis [13].

### Glucose Monitoring and Medication Adjustment

We tracked the amount and type of medications participants were taking for diabetes. Because of concern that participants in the LCK group on diabetes medication other than metformin might develop hypoglycemia (particularly if they were taking sulfonylureas) if the diet was particularly effective, these participants were asked to measure their capillary blood glucose levels in the morning (fasting) and just before dinner using a home glucose meter. The study physicians used these blood glucose levels determine whether lowering diabetes medications should be recommended due to low glucose levels over the course of the study. As a further safety precaution, participants in both groups were instructed to contact the study physicians (using a pager system) if their blood sugars ever fell below 70 mg/dL. When starting the LCK diet, we used the following medication management algorithm to reduce the risk of hypoglycemia due to the reduction in carbohydrates: metformin was continued for the duration of the study unless the participant or his/her doctor requested it be lowered, at which point the dose was cut in half or discontinued completely; sulfonylurea doses were reduced in half if the entry HbA1c was <7.5% (or discontinued if the participant was on a minimum dose); sulfonylurea were discontinued if pre-dinner glucose levels went below 110 mg/dL despite prior dose reduction; thiazolidinediones were continued for participants with starting with a HbA1c above 7% and discontinued for those with starting HbA1c below 7%.

### Measures

#### Metabolic Measures

HbA1c, low density lipoprotein (LDL) and high density lipoprotein (HDL) cholesterol, triglycerides, fasting glucose and insulin, and C-reactive protein were assessed from a fasting blood specimen at a commercial CLIA-certified laboratory (Quest Diagnostics, Madison, NJ). Subjects were weighed with no jackets or shoes using the same calibrated scale at each time. Blood pressure was measured after participants sat for 3 minutes. Two measurements were taken at least one minute apart and averaged for the analysis. HOMA was used to estimate insulin resistance by using model-derived estimates (HOMA2-IR; http://www.dtu.ox.ac.uk/homacalculator) [14].

#### Food Intake

Food intake was assessed with an online 24-hour food recall questionnaire created by the National Cancer Institute [15]. We used the Food Craving Inventory [16] to measure food cravings, defined as “an intense desire for a specific food that is difficult to resist.” We adapted the scale to provide information about cravings for carbohydrates/starches (corn bread, popcorn, rolls, biscuits, sandwich bread, rice, baked potato, pasta, French fries, and chips; α = .82) and sweets (brownies, cookies, candy, chocolate, donuts, cake, cinnamon rolls, ice cream, pancakes or waffles, and cereal; α = .88).

#### Psychological Measures

Participants completed the Diabetes Distress Scale [17], a measure of emotional upset related to having diabetes. Each item was rated on a scale ranging from 1 (*not a problem*) to 5 (*a serious problem*). (Due to a programming error the upper limit was set at 5 and not 6.) We measured depression with the 20-item Center for Epidemiologic Studies Depression Scale [CES–D; 18], which assesses aspects of depressive mood that occurred during the previous week. We also separately examined four items that tap into positive affect; higher scores reflect greater positive affect [19]. We assessed negative mood between meals by asking, “Over the past week, if it had been a few hours since you last ate, did you tend to be bothered by the following symptoms?” The items were: “nervousness or anxiety,” “depression, sad mood, or crying,” “mood swings,” “irritability,” and “anger or aggressiveness.” Responses were assessed with a 5-point scale ranging from 1 (*never*) to 5 (*very often*), with higher scores reflecting a worse mood between meals (α = .85). The emotional eating subscale of the Dutch Eating Behavior Scale [20] describes eating in response to emotions (such as anger or irritation). We used the Body Responsiveness Questionnaire to measure the importance of interoceptive awareness and perceived disconnection between psychological and physical states [21]. We assessed hunger, cognitive restraint, and disinhibited eating with the Three-Factor Eating Questionnaire [22].

#### Physical Measures

Physical symptoms were assessed with an adaptation of the Health Symptom Checklist, a short, face-valid measure of physical symptoms [23]. Participants reported on a scale from 1 (*not at all*) to 4 (*very often*) how often over the past week they had symptoms such as dizziness, shortness of breath, headaches, general aches and pains, heartburn or acid reflux, constipation, and diarrhea. We assessed physical activity using a short version of the International Physical Activity Questionnaire [IPAQ–Short; 24]. Participants were asked about three types of physical activity (vigorous, moderate and walking) over the “last 7 days.” Using both the total amount of activity and the number of activity sessions, the IPAQ categorizes participants as having low (1), moderate (2) or high (3) levels of regular physical activity.

### Statistical Analyses

The primary trial endpoint was the change in HbA1c from baseline to 3 months within each treatment arm (using a paired-samples *t*-test on the scores collected at baseline and 3 months later) and as compared to one another (using an independent-samples *t*-test for the change scores). This primary analysis was conducted on an intention-to-treat basis, including all participants who were randomly assigned and had data at both baseline and 3 months. As this was a pilot trial, we based the sample size on the resources available to perform the trial, rather on sample size calculations. To assist in planning future trials, we also calculated standardized mean differences (i.e., Cohen's d effect sizes) with 95% confidence intervals (CI) for the change from baseline to the 3-month follow-up time point between groups. Small, medium, and large effect sizes for Cohen's d have been defined as 0.2, 0.5, and 0.8, respectively [25], [26]. All statistical analyses were performed using SPSS (release 20).

## Results

**Figure 1:**
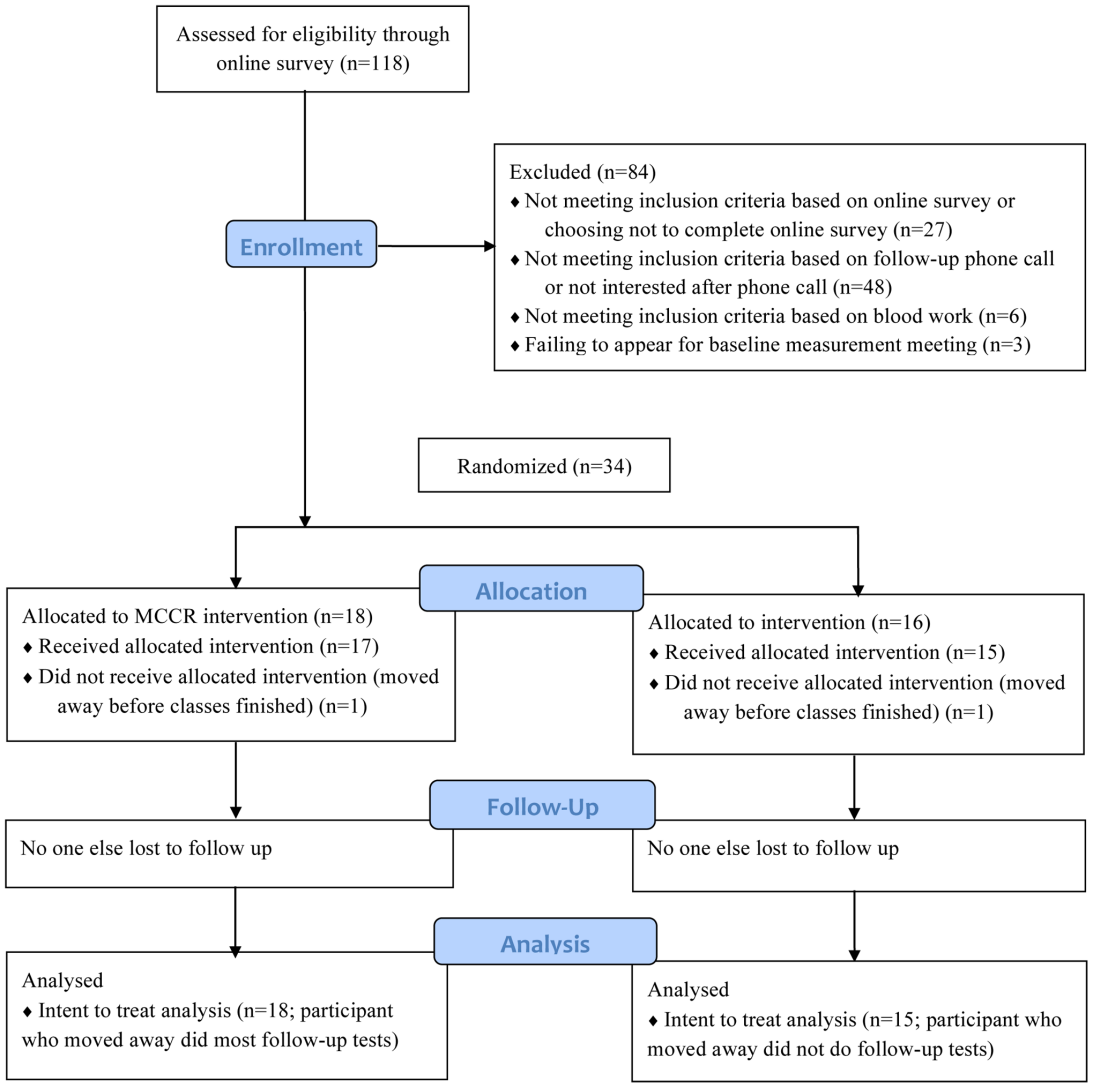
Study participant flowchart. LCK = Low carbohydrate diet group. MCCR = Medium carbohydrate, low fat, calorie-restricted, carbohydrate counting diet.

We enrolled and randomized 34 participants to the MCCR (n = 18) or LCK (n = 16) diet groups (Figure 1) in the fall of 2012 (September through November). Post-intervention data collection was completed on all but one participant (in the LCK group), who moved before classes ended. An MCCR participant also moved away, but continued to have her blood tested for the 3-month time period and thus was included in the intention-to-treat analysis for all available measures. Participant baseline characteristics are summarized in Table 1. Four participants had prediabetes (HbA1c>6.0% but <6.5% and on no diabetes medications): 3 in the MCCR group and 1 in the LCK group. Class attendance was high and similar in both groups. Excluding participants who moved away (but including any absences such as needing to work late or being ill), participants in the MCCR group participants attended an average of 86% of classes and participants in the LCK group attended an average of 89% of classes.

**Table 1:** Baseline demographic and clinical characteristics.

	LCK diet	MCCR diet
Years with diabetes	7.8 (7.5)	6.4 (4.9)
Age	64.8 (7.7)	55.1 (13.5)
White	13 (81.3%)	11 (61.1%)
Black	1 (6.3%)	1 (5.6%)
Hispanic/Latino	1 (6.3%)	2 (11.1%)
Asian/Pacific Islander	1 (6.3%)	4 (22.2%)
Female	9 (56.3%)	16 (88.9%)
No diabetes medication use	4 (25%)	5 (28%)
Use of metformin only	5 (31%)	8 (44%)
Use of metformin and another oral diabetes agent	7 (44%)	5 (28%)
BMI>30	11 (69%)	15 (83%)
Hypertension	10 (63%)	14 (78%)
Dyslipidemia	13 (81%)	10 (56%)

Numbers represent means (standard deviations) or number (percent of diet group).

BMI = body mass index.

### Changes in Diet

From baseline to 3 months, participants in the MCCR group reported reducing their energy intake by 792.1 kcal, more than the target of 500 kcal (Table 2). They reduced their reported consumption of carbohydrates from an average of 224 grams per day to 160 grams per day, near the goal of approximately 165 grams per day. Participants in the MCCR group also reported substantial reductions in fat intake, decreasing it by nearly in half by total grams per day (Table 2). Despite not having specific instruction around caloric restriction, participants in the LCK group had a similar reduction in energy intake as the MCCR group. They also reduced their reported net grams of carbohydrate consumption, to an average of 57.8 grams per day, near the target of between 20 and 50 grams per day; At three months, 57% (8/14) reported consuming 50 grams or fewer per day of carbohydrate. As a percentage of total calories, the LCK also increased their total fat consumption an average of 20%. Interestingly, actual number of grams of fat consumed per day based on diet recall essentially remained the same (decrease of 2.0 grams); the increase in proportion of calories from fat was primarily due to a decrease in carbohydrate intake. Compared to the MCCR group, the LCK group had a statistically significant greater reduction in net carbohydrate consumption, a greater reduction in percent of calories from sugar, and a greater increase in percent of calories from fat and saturated fat.

**Table 2:** Changes in diet during intervention based on 24-hour diet recall.

	LCK diet			MCCR diet			Difference between groups (LCK – MCCR)	
	0 mos	3 mos	Mean Δ, *d*	0 mos	3 mos	Mean Δ, *d*	Mean Δ, *d*	95% CI
Kilocalories	2,390.6 (1,542.7)	1,693.7 (569.1)	−696.9, −0.7	2,172.9 (784.1)	1,380.8 (527.6)	−792.1**, −1.2	95.2, 0.1	[−749.2 to 939.6]
Net carbohydrates (carbs – fiber; g)	208.9 (100.6)	57.8 (41.5)	−151.0**, −2.1	207.8 (77.3)	138.5 (54.7)	−69.3*, −1.0	−81.7*, −0.8	[−156.0 to −7.5]
Net carbohydrates (% of total Kilocalories)	38.1 (11.8)	14.4 (11.9)	−23.7**, −2.0	39.5 (12.0)	40.7 (9.3)	1.2, 0.1	−24.9**, −1.7	[−35.7 to −14.1]
Sugar (g)	78.7 (48.4)	33.1 (25.4)	−45.5**, −1.2	94.2 (57.1)	62.2 (30.4)	−32.0*, −0.7	−13.6, −0.2	[−54.9 to 27.8]
Sugar (% of total Kilocalories)	14.3 (7.4)	8.3 (7.2)	−6.0*, −0.8	17.4 (9.2)	18.5 (7.4)	1.1, 0.1	−7.1*, −0.8	[−13.9 to −0.3]
Protein (g)	115.0 (68.7)	105.7 (51.7)	−9.2, −0.2	97.0 (35.7)	67.9 (27.9)	−29.1**, −0.9	19.9, 0.3	[−24.1 to 63.8]
Protein (% of total Kilocalories)	19.9 (5.8)	24.2 (6.1)	4.3, 0.7	18.8 (7.8)	20.5 (6.8)	1.6, 0.2	2.6, 0.3	[−4.7 to 10.0]
Fat (g)	112.2 (107.9)	110.2 (40.6)	−2.0, 0.0	100.2 (68.4)	56.1 (30.1)	−44.1**, −0.9	42.1, 0.5	[−19.4 to 103.5]
Fat (% of total Kilocalories)	37.6 (11.3)	58.0 (8.6)	20.4**, 2.0	38.9 (11.2)	35.1 (8.7)	−3.9, −0.4	24.2**, 1.9	[15.1 to 33.4]
Saturated Fat (g)	37.1 (33.9)	39.7 (19.1)	2.6, 0.1	31.4 (16.6)	16.3 (7.7)	−15.1**, −1.2	17.7, 0.7	[−1.1 to 36.5]
Saturated Fat (% of total Kilocalories)	12.8 (4.6)	21.0 (7.8)	8.2**, 1.3	12.9 (5.1)	10.7 (3.6)	−2.3, −0.5	10.5**, 1.4	[5.2 to 15.8]

Means and standard deviations; means exclude participants without follow-up data; *d* = Cohen's *d*; CI = Confidence Interval;

* = *p*<0.05, and

** = *p*<0.01 for *t*-tests within groups (Mean Δ columns for each of the diets) and between groups (Mean Δ column for the difference column).

Participants in the LCK group reported on their beta-hydroxybutyrate levels as measured by home monitoring on blood ketone test strips. At week 4, when most participants should have transitioned into nutritional ketosis, 55% (6/11) reported a level of at least 0.5 mM and 73% (8/11) reported a level of at least 0.3 mM. By week 6, 75% (9/11) reported a level of at least 0.5 mM and 92% (11/12) reported a level of at least 0.3 mM.

### Changes in Clinical and Laboratory Outcomes

The mean HbA1c did not change during the trial in the MCCR group, but decreased by −0.6% in the LCK group (*p* = 0.04, Table 3). The Cohen's d for HbA1c change in the LCK group was −1.8, a very large effect size. No one in the MCCR group achieved a normal HbA1c of <5.7%, compared to 13% percent (n = 2) of the LCK group. All individuals in the LCK group showed a drop in HbA1c, whereas 72% (n = 13) of the participants in the MCCR group showed an improved HbA1c (χ^2^ = 5.2, *p* = 0.02). More specifically, 56% (n = 9) of participants in the LCK group showed a clinically significant drop of 0.5% or greater in HbA1c, whereas only 22% (n = 4) of participants in the MCCR group showed a drop of 0.5% or greater of HbA1c (χ^2^ = 4.2, *p* = 0.04).

**Table 3:** Changes in clinical, laboratory, and psychological outcomes during intervention.

	LCK diet			MCCR diet			Difference between groups (LCK – MCCR)	
	0 mos	3 mos	Mean Δ, *d*	0 mos	3 mos	Mean Δ, *d*	Mean Δ, *d*	95% CI
HbA1c (%)	6.6 (0.3)	6.0 (0.3)	−0.6**, −1.8	6.9 (0.7)	6.9 (1.1)	0.0, 0.0	−0.6*, −0.8	[−1.1 to −0.03]
Weight (kg)	100.1 (26.4)	94.6 (23.3)	−5.5**, −0.2	99.7 (24.2)	97.1 (23.3)	−2.6*, −0.1	−2.9, −0.6	[−6.3 to 0.5]
BMI (kg/m^2^)	36.2 (8.2)	34.3 (7.4)	−1.9**, −0.2	37.4 (6.4)	36.4 (6.4)	−0.9*, −0.1	−1.0, −0.6	[−2.2 to 0.2]
Systolic BP (mm Hg)	130.7 (10.5)	136.0 (9.8)	5.3, 0.5	129.5 (13.0)	134.2 (11.8)	4.7, 0.4	0.6, 0.0	[−8.8 to 10.0]
Diastolic BP (mm Hg)	76.3 (6.8)	78.9 (6.5)	2.5, 0.4	79.9 (12.2)	80.2 (7.3)	0.3, 0.0	2.2, 0.3	[−4.4 to 8.9]
LDL (mmol/l)	89.2 (25.8)	87.1 (33.6)	−2.1, −0.1	98.5 (24.7)	95.1 (21.6)	−3.4, −0.1	1.3, 0.1	[−12.3 to 15.0]
HDL (mmol/l)	50.1 (14.2)	51.0 (14.8)	0.9, 0.1	46.9 (11.0)	46.0 (11.3)	−0.9, −0.1	1.8, 0.3	[−2.5 to 6.2]
Triglycerides (mg/dL)	123.6 (61.3)	101.3 (39.9)	−22.3, −0.4	172.2 (74.4)	168.3 (88.1)	−3.9, 0.0	−18.4, −0.3	[−57.3 to 20.5]
Fasting Glucose (mg/dL)	124.4 (28.3)	113.3 (15.3)	−11.1, −0.5	140.6 (34.3)	139.5 (47.4)	−1.2, 0.0	−9.9, −0.3	[−36.2 to 16.4]
Fasting Insulin (µIU/mL)	12.2 (10.0)	9.3 (4.8)	−2.9, −0.4	10.1 (4.4)	11.1 (4.9)	1.0, 0.2	−3.9, −0.6	[−8.2 to 0.5]
C-Reactive Protein (mg/dL)	7.3 (7.5)	5.8 (5.4)	−1.5*, −0.2	4.4 (3.6)	3.8 (3.8)	−0.7*, −0.2	−0.9, −0.5	[−2.1 to 0.4]
HOMA2-IR	1.7 (1.3)	1.3 (0.6)	−0.4, −0.4	1.5 (0.6)	1.6 (0.8)	0.1, 0.1	−0.5, −0.6	[−1.1 to 0.1]
Heartburn or Acid Reflux	1.8 (1.0)	1.1 (0.3)	−0.7*, −1.2	1.4 (0.6)	1.4 (0.7)	0.0, 0.0	−0.7*, −0.9	[−1.3 to 0.1]
Carbohydrate cravings	2.1 (0.8)	1.4 (0.6)	−0.6**, −0.9	2.1 (0.6)	1.8 (0.6)	−0.3*, −0.5	−0.3, −0.5	[−0.8 to 0.1]
Sweet cravings	2.1 (0.7)	1.4 (0.5)	−0.6**, −1.1	2.4 (0.9)	2.1 (0.8)	−0.2*, −0.3	−0.4, −0.6	[−0.9 to 0.0]
Diabetes Distress	1.8 (0.5)	1.3 (0.6)	−0.5**, −0.9	2.3 (0.9)	2.1 (0.8)	−0.2, −0.2	−0.3, −0.4	[−0.8 to 0.2]
Physical Activity (IPAQ)	1.7 (0.5)	1.9 (0.5)	0.3, 0.4	1.9 (0.8)	2.1 (0.7)	0.2, 0.2	0.0, 0.1	[−0.3 to 0.4]
CES-D Depression	10.6 (8.2)	9.2 (10.3)	−1.4, −0.1	12.0 (8.3)	10.8 (10.0)	−1.2, −0.1	−0.2, 0.0	[−5.3 to 4.9]
CES-D Positive Affect	9.5 (2.7)	9.5 (2.7)	0.1, 0.0	8.9 (2.4)	9.6 (2.7)	0.7, 0.3	−0.6, −0.4	[−1.8 to 0.6]
Negative mood between meals	1.8 (0.6)	1.3 (0.5)	−0.5**, −0.9	1.8 (0.7)	1.6 (0.7)	−0.2, −0.2	−0.3, −0.6	[−0.8 to 0.1]
Emotional Eating	2.6 (0.9)	2.0 (0.9)	−0.6*, −0.7	2.8 (1.0)	2.4 (0.8)	−0.4*, −0.5	−0.1, −0.2	[−0.7 to 0.4]
Interoceptive Awareness	3.6 (1.4)	4.3 (1.3)	0.7, 0.5	4.0 (1.7)	4.4 (1.7)	0.3, 0.2	0.4, 0.2	[−.09 to 1.6]
Perceived Disconnection	3.6 (1.2)	2.7 (1.2)	−0.9*, −0.7	3.8 (1.2)	3.5 (1.6)	−0.3, −0.2	−0.5, −0.4	[−1.5 to 0.5]
TFEQ Hunger	16.8 (2.6)	14.0 (2.5)	−2.8**, −1.1	18.2 (4.2)	16.2 (3.9)	−2.1**, −0.5	−0.7, −0.3	[−2.6 to 1.1]
TFEQ Dietary Restraint	20.0 (3.2)	23.8 (4.7)	3.8*, 1.0	19.3 (3.4)	24.1 (4.4)	4.8**, 1.2	−1.0, −0.2	[−4.4 to 2.5]
TFEQ Disinhibition	21.9 (3.0)	17.7 (3.2)	−4.1**, −1.3	23.4 (4.0)	21.3 (4.9)	−2.1*, −0.5	−2.0, −0.6	[−4.6 to 0.6]

Means and standard deviations; means exclude participants without follow-up data; *d* = Cohen's *d*; CI = Confidence Interval;

* = *p*<0.05, and

** = *p*<0.01 for *t*-tests within groups (Mean Δ columns for each of the diets) and between groups (Mean Δ column for the difference column). See Methods for details of psychological scales.

In the LCK group, 44% (n = 7) discontinued one or more oral diabetes medications, compared to LCK group 11% (n = 2) of the MCCR group (χ^2^ = 4.6, *p* = 0.03; Table 4). Seven patients in the LCK group discontinued diabetes medications: 5 (31%) discontinued a sulfonylurea (glyburide or glipizide), 2 (13%) discontinued a dipeptidyl peptidase −4 inhibitor (sitagliptin), and 2 (13%) discontinued metformin; some participants discontinued more than one class of diabetes medication. Two patients in the MCCR group discontinued at least one diabetes medication: 1 (5%) discontinued a sulfonylurea (glipizide) and 1 (5%) discontinued metformin. Thus, 31% discontinued sulfonylureas in the LCK group, compared to 5% in the MCCR group (χ^2^ = 3.8, *p* = 0.05).

**Table 4:** Medication use at baseline and 3 months among participants taking diabetes medication at baseline.

Participant	0 months daily dose	3 months daily dose
LCK diet		
1	Glimepiride 4 mg	Unknown (dropped out of study)
	Actos 15 mg	
	Exenatide 5 mg twice a day	
	Metformin 1000 mg twice a day	
2	Metformin 500 mg twice a day	No change
3	Metformin 850 mg twice a day	No change
4	Metformin 1000 mg twice a day	No change
5	Metformin 2000 mg	No change
6	Metformin 500 mg	Metformin discontinued
7	Glyburide 2.5 mg twice a day	Glyburide and Metformin discontinued
	Metformin 1000 mg twice a day	
8	Glipizide 2.5 mg	Glipizide discontinued
	Metformin 1000 mg twice a day	
9	Glipizide 5 mg	Glipizide discontinued
	Metformin 1000 mg twice a day	
10	Glyburide 2.5 mg twice a day	Glyburide discontinued
	Metformin 500 mg	
11	Januvia 50 mg	Januvia discontinued
	Metformin 1000 mg twice a day	
12	Glyburide 2.5 mg	Glyburide and Januvia discontinued
	Januvia 100 mg	
	Metformin 1000 mg twice a day	
MCCR diet		
1	Metformin 500 mg	No change
2	Metformin 500 mg twice a day	No change
3	Metformin 500 mg twice a day	No change
4	Metformin 500 mg twice a day	No change
5	Metformin 500 mg twice a day	No change
6	Metformin 1000 mg twice a day	No change
7	Metformin 1000 mg twice a day	No change
8	Glipizide 10 mg	No change
	Metformin 1000 mg twice a day	
9	Glimepiride 8 mg	No change
	Januvia 1000 mg twice a day	
	Metformin 50 mg twice a day	
10	Glipizide 2.5 mg twice a day	No change
	Metformin 1000 mg twice a day	
11	Glipizide 5 mg	No change
	Metformin 2000 mg	
	Januvia 50 mg	
12	Metformin 850 mg 3 times a day	Metformin lowered to 500 mg twice a day
13	Glipizide 5 mg	Glipizide discontinued
	Metformin 500 mg twice a day	
	Acarbose 50 mg three times a day	

**Figure 2:**
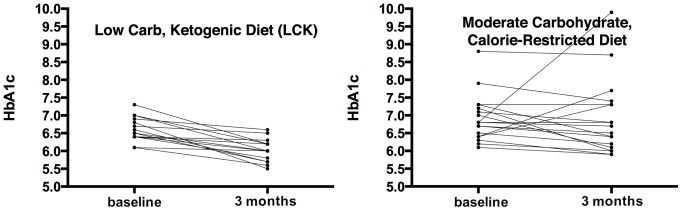
Change in HbA1c by diet group. Both panels show individual lines for the course of HbA1c from baseline to 3 months after intervention initiation for each trial participant. The left panel displays this data for the low carbohydrate diet group (mean change −0.6%), while the right presents the data for the moderate carbohydrate diet group (mean change 0%). See text for further discussion.

**Figure 3:**
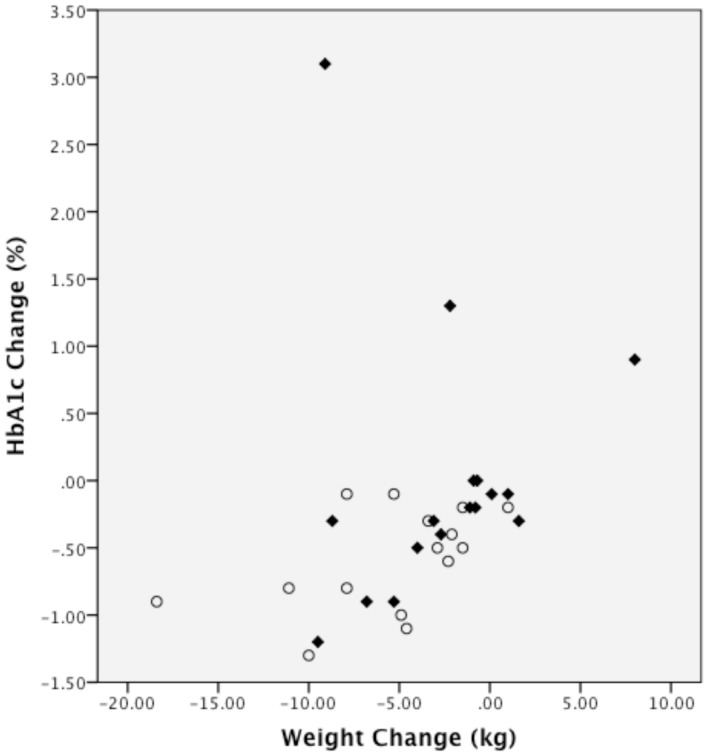
Relationship between change in hemoglobin A1c and change in weight. The figure plots the change in HbA1c vs. the change in weight from baseline to 3 months for each individual (Spearman rank correlations: LCK = 0.45, *p* = 0.096; MCCR = 0.47, *p* = 0.055). The LCK group is shown as open circles; the MCCR group is shown as diamonds.

One participant in the MCCR group increased their HbA1c substantially (3.1%; see Figure 2). When we repeated the analysis for change in HbA1c excluding this participant, results remained similar: within the MCCR group, HbA1c change was not significant (−0.2%, *p* = .19); the between group difference of change in HbA1c (−0.4%) favored the LCK group (95% CI, −0.8% to −0.02%, *p* = 0.04). Additionally, we examined the correlation between change in HbA1c and change in weight, but this did not reach statistical significance (Spearman rank correlations: LCK = 0.45, *p* = 0.096; MCCR = 0.47, *p* = 0.055; Figure 3).

**Figure 4:**
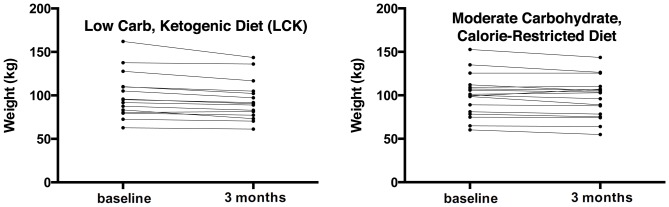
Change in weight by diet group. Both panels show individual lines for the course of weight from baseline to 3 months after intervention initiation for each trial participant. The left panel displays this data for the low carbohydrate diet group (mean change −5.5 kg), while the right presents the data for the moderate carbohydrate diet group (mean change −2.6 kg).

**Figure 5:**
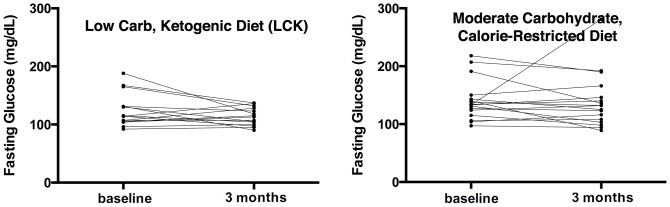
Change in fasting glucose by diet group. Both panels show individual lines for the course of fasting glucose from baseline to 3 months after intervention initiation for each trial participant. The left panel displays this data for the low carbohydrate diet group (mean change −11.1 mg/dL), while the right presents the data for the moderate carbohydrate diet group (mean change −1.2 mg/dL).

**Figure 6:**
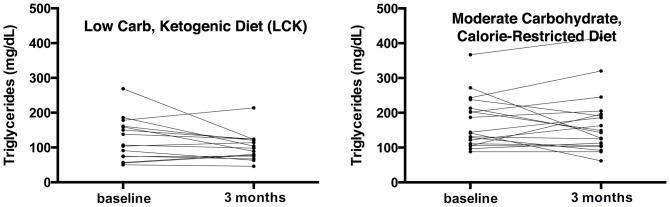
Change in triglycerides by diet group. Both panels show individual lines for the course of triglycerides from baseline to 3 months after intervention initiation for each trial participant. The left panel displays this data for the low carbohydrate diet group (mean change −22.3 mg/dL), while the right presents the data for the moderate carbohydrate diet group (mean change −3.9 mg/dL).

Both groups had significant weight loss (Figure 4). However, the mean weight loss tended to be larger in the LCK group (MCCR: −2.6 kg and −2.8% of body weight; LCK: −5.5 kg and −5.5% of body weight), but the difference was not statistically significant at *p* = 0.09. The mean C-reactive protein also declined in both groups (Table 3). No statistically significant changes occurred for blood pressure, glucose (Figure 5), lipids (Figure 6), insulin, or HOMA2-IR.

We examined whether any reported physical symptoms changed. Compared to the MCCR group, the LCK group had a greater reduction in heartburn, reported as frequency of symptoms over the past week (Table 3). Within the LCK group, participants experienced increased constipation (mean change of 0.4, *p* = 0.03) as well as decreased general aches and pains (mean change of −0.8, *p* = 0.003). There were no other statistically significant changes in physical symptoms.

### Changes in Psychological and Physical Outcomes

Participants within both groups reported significantly reduced carbohydrate and sweet cravings, emotional eating, hunger, and eating disinhibition but increased dietary restraint (Table 3). When the two groups were compared there were no statistically significant differences in changes in these measures between groups. There were no statistically significant changes in depressive symptoms and positive affect within either group, nor were there statistically significant differences in changes in mood between groups. In the LCK group, participants reported statistically significant decreases in diabetes distress, negative mood between meals, and perceived disconnection between psychological and physical states. As each of these measures also tended to decline in the MCCR (though without statistically significant declines), comparisons of change in these measures between groups did not show statistically significant changes.

## Discussion

A key finding of this randomized, controlled trial was that a low carbohydrate diet was more effective than a standard, moderate carbohydrate diet at reducing HbA1c at three months, our primary outcome point. These results are consistent with those of several prior studies [5], which have found substantial improvements in glycemic control with low carbohydrate diets in the setting of a metabolic ward [27], or in uncontrolled studies [28]. These results provide important support for the benefit of low carbohydrate diets in type 2 diabetes for glycemic control, as well as the feasibility of adhering to the diet for at least three months in a community setting. In addition, the improvement in glycemic control was observed despite greater decreases in diabetes medications, particularly sulfonylureas, in the LCK group. This combination of findings suggests another possible benefit of a low carbohydrate diet intervention that may warrant further investigation. Of note, we observed no episodes of clinically evident hypoglycemia in our study, though any reassurance this might provide is substantially limited by the small numbers and short duration of study. However, our findings suggest a low carbohydrate diet may hold promise as a strategy to simultaneously improve glycemic control while allowing discontinuation of medications most likely to cause serious hypoglycemia.

Another important finding was the consistency with which the low carbohydrate diet improved glycemic control. All individuals in the LCK group showed a decrease in HbA1c, and this was a statistically significant advantage compared to the proportion of persons in the MCCR group who decreased their HbA1c. The proportion of persons achieving a decrease in HbA1c of at least 0.5% was more than twice as high in the LCK (56% vs. 22%), which again was statistically significant. As this is a modest sized study, this effect needs further confirmation in a larger trial with longer follow-up. However, the idea that a low carbohydrate diet may be more consistently effective than other diets is supported by recent research on the interaction of insulin-resistance status with response to low fat versus low carbohydrate diets. For women assigned to a low carbohydrate diet, degree of insulin resistance was not significantly associated with dietary adherence or weight loss. For women assigned to a low fat diet however, if they were insulin resistant they were less likely to lose weight or adhere to the diet [29]. Thus, individual variability in responses to the low carbohydrate diet may be more constrained in persons with significant insulin resistance than variability in responses to low fat or moderate low carbohydrate diets.

An important concern with low carbohydrate diets that increase the proportion of calories from fat is that this will have adverse effects on lipids. Although the LCK diet group reported an increased percentage of dietary fat intake, the reported total quantity of fat intake did not increase; the increased percentage of fat intake occurred because carbohydrate intake decreased. Despite the LCK diet's relatively high fat content (rising from 38% to 58% of calories from fat, as compared to MCCR's reduction from 39% to 35% of calories from fat), significant elevations in LDL were not observed on the LCK diet. These results suggest that in persons with diabetes, a very low carbohydrate diet has effects that are neutral and even beneficial, on average, on lipids. These results are similar to previous research, which found that individuals on a LCK versus a low glycemic index diet had greater reductions in HbA1c and weight as well as beneficial effects on blood lipids [28]. This previous research was limited, however, by low retention. Our results also need to be interpreted with some caution in this regard as the sample size and duration of follow-up were both limited.

Both groups had significant weight loss, even though the LCK group tended to lose more weight that the MCCR group (although only significant to the *p* = 0.09 level), even though only the MCCR group aimed to restrict calories. This overall finding, while not statistically significant, is consistent with some prior diet and weight loss studies. Several well-designed randomized, controlled trials have found similar or greater weight loss with low carbohydrate diets that do not calorie restrict, compared to higher carbohydrate diets that focus on calorie restriction [30]. We found a marginally significant correlation between change in HbA1c and change in weight. Other studies of low carbohydrate diets in diabetes have also found evidence of improvements in HbA1c that are not closely correlated to changes in body mass [28] [31]. This suggests that weight loss alone is not driving the improvement in HbA1c during low carbohydrate diets.

We found a statistically significant reduction in the LCK group for poor mood (such as mood swings and irritability) if it had been a few hours since they had last eaten; there was a slight decrease in this measure in the MCCR group, but it was not statistically significant. While the trend toward greater improvement in this measure with a LCK must be viewed cautiously given the lack of a statistically significant difference, it is possible that this mood stability is due to steadier blood sugar in the LCK group, as blood sugar variability has been found to be related to poor mood [32]. Other mood effects did not seem to show statistically significant differences or even clear trends, between groups. Both groups were less likely to eat when upset (emotional eating), which may be related to the psychological tools to support behavior change that both groups received.

There are several important limitations of our study. An important limitation is that this trial was designed with primary outcomes at 3 months, although we aim to continue follow-up to 12 months. Larger scale trials with longer-term follow-up are clearly needed to better address the long-term feasibility of follow a low carbohydrate diet for type 2 diabetes, as well better establishing the long-term outcomes and possible adverse effects. We did not stratify randomization by sex. Due to chance, about 56% of the LCK group was female compared to 89% of the MCCR group. This could possibly have affected our results, but adjustments for sex did not dramatically alter our primary outcome (*p* = .06 when assessing change in HbA1c). We included both individuals receiving diabetes medications or not on medication, but we excluded people taking insulin, so our results may not extend to individuals using insulin. We included a small group of individuals with prediabetes. While we believe that extending the results to persons with prediabetes requires further study due to the small numbers studied, the inclusion of persons with prediabetes likely made it more difficult to detect a difference in HbA1c between diet groups because we included individuals with more limited room for improvement than individuals with frank type 2 diabetes. Finally, as in most similar studies, we collected dietary intake data before and after randomization to the two diet groups. While we and our participants made an earnest effort to collect these data, there is a rich literature indicating that there are important inaccuracies this data that limit our ability to relate specific changes in self-reported diet content to outcomes [33]. While our diet data are reported in Table 2, at least 20% of daily energy intake is “missing” at baseline. It is well established that the typical ambulatory adult expends between 30–35 kcal per kg daily. Our participants at baseline (body weight about 100 kg) should thus have required 3000–3500 kcal daily to be in energy balance, yet they reported 2200–2400 kcal of daily intake. Furthermore, at 3 months our subjects reported daily energy deficits of about 700 kcal per day, whereas their weight losses (particularly in the MCCR group) reflected a substantially smaller energy deficit. We thus believe the dietary intake data are likely to reflect in part what participants understood we wanted to hear. Due to concerns about reporting bias in a study in which the subjects knew that we were contrasting carbohydrate intakes, we did not try to assess a quantitative relationship between self-reported dietary carbohydrate intake and outcomes such as weight loss.

Despite these limitations, our data suggest that, in overweight and obese individuals with type 2 diabetes, a very low carbohydrate, high fat, non calorie-restricted diet may be more effective at improving blood glucose control than a medium carbohydrate, low fat, calorie-restricted, carbohydrate counting diet that remains the standard for most diabetes education efforts. The most recent dietary guidelines by the ADA state that the “long-term metabolic effects of very low-carbohydrate diets are unclear” [34]. In addition we show that psychological intervention can significantly reduce carbohydrate and sweet cravings, emotional eating, hunger, and eating disinhibition as well as increase dietary restraint irrespective of the diet. In light of the promising results we observed in improved glycemic control while reducing medications that elevate the risk of hypoglycemia, we believe this important concern about the long-term effects of low carbohydrate diets highlights the importance of larger randomized clinical trial testing the effectiveness and safety of low carbohydrate diets for individuals with type 2 diabetes over sustained periods of time.

## Supporting Information

Checklist S1 **CONSORT Checklist.**
(PDF)

Protocol S1 **Study Protocol.**
(PDF)
