# Supplementary material for: A Randomized Pilot Trial of a Moderate Carbohydrate Diet Compared to a Very Low Carbohydrate Diet in Overweight or Obese Individuals with Type 2 Diabetes Mellitus or Prediabetes
Source: PLoS One. 2014 Apr 9;9(4):e91027. doi: 10.1371/journal.pone.0091027 (PMC3981696; doi:10.1371/journal.pone.0091027)
Supplement: Protocol S1 — Study Protocol. (PDF) [file pone.0091027.s002.pdf]

Trial Protocol:

Procedures

Recruitment

- Participants will be recruited from the community by newspaper and online advertisements and notices, advertisements posted in public places, etc. (see Recruitment section for more details.
- Initial study contact and screening: Advertisements will direct interested people to the study website, which will describe study participation in further detail, including what is involved in participating in the group classes, as well as the assessment visits; visitors will be instructed to read the full-study consent form. Interested people will be asked to sign an online consent to complete a screening survey to assess study eligibility; after signing this consent, they will complete the online screening survey.
- Those who do not pass the online screener will be thanked for their time and told that they are not eligible. Those who pass the online screener will be asked to provide contact information so that study staff can reach them to review eligibility and discuss next steps.
- During this phone screen, study staff will confirm potential interest and eligibility, and answer any questions the participant may have about the study. The goal of this phone contact is to a) ensure that it is likely that a participant will be eligible before scheduling an in-person interview and b) ensure that participants understand the commitment involved in study participation.
- Those who meet initial criteria and express continued interest in participating will schedule a consent visit for additional screening, including blood draw to confirm eligibility.

Consent and screening visit. Participants will come to the Osher Center for the first part of the initial visit (a-f, below) and then a Quest lab for the second part of the initial visit (g, below). In total, this will take approximately 1 hour. The following steps will be included in the visit:
a. Informed consent: Informed consent will be obtained from all study participants before performing any study procedures. Study staff will provide a copy of the Experimental Subjects Bill of Rights, then review the consent and HIPAA forms with the participant and answer any questions. Those who are interested will be asked to sign the forms. The original will be kept by the study staff in a locked filing cabinet; they will not have participant ID numbers on them.
b. Diabetes and Health History Interview: Study staff will conduct a brief medical history to collect data on diabetes history, comorbid conditions and medication use. This procedure also screens for medical, substance abuse, or mental health conditions that might interfere with the participants' ability to participate in the study.
c. Authorization to release information: Because it is important for study staff to communicate with the usual care team, we will ask participants to sign an authorization to release information form. This will allow study staff to communicate with the

participant's usual care medical team regarding changes in medication, results of any lab tests, and any other pertinent information regarding health.
d. Weight and height: To confirm eligibility, we will measure Body Mass Index (BMI). Height and weight measurements will be assessed while the participant is dressed in a gown. Body height will be measured to the nearest 0.1cm. Body mass index is calculated as weight (kg) divided by height squared ($meters^2$). In the event that someone is found to be ineligible based on BMI the reasons why will be explained and study participation will end. If the participant meets the eligibility criteria after the above assessments, the remaining procedures will be performed. Waist circumference may also be measured.
e. Blood pressure and heart rate: Blood pressure and heart rate will be measured using an automated blood pressure and heart rate monitor. Other vital signs such as temperature and respiration may be measured.
f. Collection of Saliva for genetics: Participants will be asked to salivate into a saliva collection kit for genetic material. This process takes no more than about 5 minutes and is noninvasive. One the containers are closed, the genetic material is shelf stable for several years.
g. Fasting Blood Draw: A fasting blood draw will be performed to assay hemoglobin A1c (HBA1c), thyroid stimulating hormone, and a metabolic panel for liver and kidney function. These results will be used to determine study eligibility. Subjects will be asked to refrain from alcohol use and strenuous exercise for 24 hours prior to blood sampling. Specimens will be identified by participant ID only. (Done at a Quest lab.)

Contact with usual care medical team. After the initial meeting with participants (a-f, above), we will contact the usual care medical team about the fact that one of their patients would like to join our study. We will describe the research and invite the medical team to contact us with any questions or concerns.

Notification of eligibility. In the event that participants are not found to meet all eligibility criteria, the reasons why participants do not meet inclusion criteria for the study will be explained and study participation will end. We anticipate that this will occur infrequently after the consent visit, and will be primarily due to Hba1c<6.0 on testing performed at the consent visit. Participants will be notified when the results of their fasting blood draw are available; if these labs are in range, participants will be told that they are eligible for the study, and that they will receive an email with a link to their baseline online questionnaires approximately 3 weeks prior to the class start date. Participants will be mailed copies of their lab results if they choose. Participants will be eligible regardless of if they have home access to computers for the online questionnaires. Those without access will be able to schedule time to access the questionnaires for free using computers at the Osher Center.

Baseline Questionnaire. After completing the in-person consenting and screening visit, participants eligible for the study and still interested in enrolling will complete an online survey. Study staff will email a link to the online questionnaires approximately 3 weeks

before the class start date. They will take approximately 40-60 minutes to complete and will be about demographics, food intake, personality, mood, emotions, stress, health behaviors, and physical and emotional well-being. If someone prefers, they can arrange a time with study staff to UCSF to complete these questionnaires.

Baseline blood draw. After completing the online survey, participants will have a (fasting) blood draw to measure glucose, insulin, and cholesterol/lipids. To reduce participant burden, this blood draw will be done at a Quest lab in the community that is most convenient to a participant. Study staff will mail a lab slip for this blood draw. Participants will be required to complete this blood draw before randomization.

Randomization. After all of the above baseline assessments are complete, participants will be eligible to be randomized to study groups. Participants will complete a short online questionnaire to confirm interest and availability for the study classes and follow-up assessments, and then will indicate whether they wish to be randomized. At the end of the survey they will be e-mailed which class I have been assigned to. An online randomization provides a computerized group assignment that cannot be altered by study staff and the group assigned is immediately recorded. Randomization will be done approximately 1-2 weeks prior to the start of classes in order to minimize post-randomization drop-out due to life circumstances that might affect participant ability to attend classes (e.g. such as job loss/change, family emergency, etc.).

Intervention classes. Both groups will meet for 15 evening sessions over about 6 months. There will be 12 weekly classes, then 3 biweekly classes. The last 3 sessions are spaced out over time in order to prevent relapse of old eating patterns and weight gain and further reinforcement of the mindfulness and positive emotion practices. These final 3 sessions also help participants integrate what they have learned, understand what they have done to make changes, and recognize how to continue making progress. There will be approximately 15 participants in each class. The mindfulness and positive emotion curriculum will be similar in both groups and will be based on the SHINE study mindfulness-based intervention that we have been doing for the past several years, along with positive emotion tools that Judy Moskowitz has successfully piloted in studies with individuals with HIV and cancer. The mindfulness-based curriculum will focus on the following elements: training on topics such as mindful meditation, mindful eating, awareness of fullness and hunger signals, and taste satiety. The positive emotion curriculum will include: training on topics such as noticing and savoring positive events, gratitude, personal strengths, goals, acts of kindness, and positive reappraisal.

The primary difference between the two intervention groups will be in the diet approach. Participants will receive weekly meetings consisting of dietary counseling (low carbohydrate - LC or American Diabetes Association - ADA), emotion regulation strategies, and mindful eating skills. Patients will be encouraged to exercise at least three times per week on their own, practice the coping skills, and practice mindful

eating. Participants will be supplied with an over-the-counter magnesium supplement and, in the LC group only, be supplied with strips for measuring urinary ketones (to assess nutritional ketosis). For the stress coping and mindfulness aspect of the intervention, participants will receive handouts developed from our previous research.

Participants in the LC group will be instructed to follow a low carbohydrate diet, with carbohydrate coming from low glycemic vegetables (carbohydrate intake 10-50 grams/day not including grams of fiber). Foods permitted include meats, poultry, fish, eggs, hard cheese, green leafy vegetables, and most other non-starchy vegetables. Because most individuals self-limit caloric intake, no calorie restriction is recommended. We will use handouts from several lay-press books as an instructional guide: The New Atkins for a New You, Atkins Diabetes Revolution, and The Art and Science of Low Carbohydrate Living. For participants in the LC group, they may be asked to test their urine for ketone levels using provided ketone urine-test strips. If they do not test positive for urinary ketones, participants may be asked to test their blood using a portable glucometer which also allows the measurement of ketones.

Participants in the ADA diet group will receive standardized advice about appropriate foods. The diet includes high-fiber foods (such as vegetables, fruits, whole grains, and legumes), low-fat dairy products, fresh fish, and foods low in saturated fat. The classes will be audiotaped in order to ensure adherence by the teachers to the assigned intervention. To help with maintenance of weight loss, following the last class, participants may receive suggestions, reminders and ideas for continued and maintained weight loss approximately two times a month.

<u>Consultations with study doctors</u>. Study doctors will review the participants' medication use and blood sugars. They may recommend that the participants reduce their diabetes or blood pressure medications. The participants may be asked to schedule a time to meet with the study doctor to discuss this. These consultations will typically take place before class. If the participants have symptoms or concerns that they want to discuss with the study doctor, they can schedule a time with study staff to do this. For urgent concerns, participants may page one of the study doctors.

Continued contact with usual care medical team: When any lab results from the research or medical changes are suggested by the study doctors, we will contact the usual care medical team. We will invite the team to contact us with any concerns or questions and invite them to work with us to provide better care for the patient.

<u>Online surveys</u>. For participants without computer access and/or who prefer to do the online surveys at the Osher Center, we will schedule a time for them to come into our offices to complete any online surveys.

Interviews, questionnaires, and/or surveys.

Weekly surveys: During the intervention phase, participants will complete weekly surveys about their mood, symptoms and adherence to the diet and study practices. These measures will be completed online via Qualtrics; participants will be sent text messages reminding them to complete these measures in a timely manner. If these have not been completed by the next class session, participants will be asked to complete them at the class. (Attached)

Online diet assessments: At several points throughout the study, participants will be asked to complete the ASA24. The ASA24 assesses food intake over the prior 24-hour period. It takes approximately 20-60 minutes to complete. So that participants do not change food intake specifically for this assessment, they will not be told exactly when hey will be asked to complete ASA-24. Depending on participant preference, they will receive I will tell study staff the phone number and best times I can be reached at to be alerted to complete this.

3, 6, and 12 month follow-up visits: These 1-2 hour visits will take place approximately 3, 6, and 12 months after the class start date at the Osher Center (a and b, below), a Quest Lab (c, below), and online (d and e, below). The following procedures, previously completed at the baseline visit, will be repeated at this visit:
a. Diabetes and Health History Interview: Study staff will review current medication use, diabetes symptoms and new comorbid conditions with the participant.
b. Weight, blood pressure and heart rate.
c. Fasting Blood Draw to measure insulin, glucose, HBA1c, lipids panel, and C-reactive protein.
d. Questionnaires on Qualtrics: Participants will complete personality, mood and behavior questionnaires administered via Qualtrics. The questionnaires are projected to take approximately 20-40 minutes; participants may complete some of these questionnaires at home prior to the visit if they choose.

If a participant moves out of the Bay Area or otherwise is unable or unwilling to come for a follow-up study visit at 3, 6, or 12 months, study staff will work with them to complete questionnaires online and have a fasting blood draw at a Quest lab in their community. This blood draw would be only for the labs that have previously been done by Quest Labs (i.e. cholesterol, fasting glucose, etc.). Participants who are not willing or able to do that will be asked whether they are willing to respond to an email or phone call inquiry at each of the remaining time points about their current diet, diabetes symptoms, and weight.
